# Association between salt intake and blood pressure among community-dwelling older adults based on their physical frailty status

**DOI:** 10.1038/s41440-024-02066-y

**Published:** 2025-01-17

**Authors:** Hiroko Yoshida, Mai Kabayama, Michiko Kido, Kayo Godai, Yuya Akagi, Yaya Li, Hiroshi Akasaka, Yoichi Takami, Saori Yasumoto, Madoka Ogawa, Takeshi Nakagawa, Kazunori Ikebe, Yasumichi Arai, Yukie Masui, Takumi Hirata, Yasuyuki Gondo, Koichi Yamamoto, Kei Kamide

**Affiliations:** 1 Division of Health Sciences, Graduate School of Medicine, Osaka University, Osaka, Japan; 2 Graduate School of Nursing, Osaka Metropolitan University, Osaka, Japan; 3 Department of Geriatric and General Medicine, Graduate School of Medicine, Osaka University, Osaka, Japan; 4 Department of Hygiene and Preventive Medicine, Iwate Medical University School of Medicine, Iwate, Japan; 5 Office of International Exchange, Graduate School of Human Sciences, Osaka University, Osaka, Japan; 6 Department of Clinical Thanatology and Geriatric Behavioral Science, Graduate School of Human Sciences, Osaka University, Osaka, Japan; 7 Department of Prosthodontics, Gerodontology and Oral Rehabilitation, Graduate School of Dentistry, Osaka University, Osaka, Japan; 8 Center for Supercentenarian Medical Research, Keio University School of Medicine, Tokyo, Japan; 9 Human Care Research Team, Tokyo Metropolitan Institute for Geriatrics and Gerontology, Tokyo, Japan

**Keywords:** Salt intake, Aged, Frailty, Independent living

## Abstract

The effects of salt reduction and adequate nutrition intake among older adults with physical frailty remain controversial. Therefore, the present study investigated whether the association between daily salt intake and blood pressure among community-dwelling older adults, including the very old people, based on their physical frailty status. This cross-sectional study used data from the SONIC study, a cohort study on older adults, collected between 2010 and 2012. Daily salt intake was estimated from the brief self-administered diet history questionnaire. Participants were stratified by groups based on the use of antihypertensive medication and their physical frailty status. There were 1975 participants with an average age of 76.5 ± 6.5 years and 53.1% were female. No association was observed between daily salt intake and blood pressure among participants with physical frailty regardless of the use of antihypertensive medication. In contrast, an association was noted between daily salt intake and systolic blood pressure among the robust without antihypertensive medication group (*β* = 0.08, *p* = 0.038), and the odds ratios for systolic blood pressure ≥140 mmHg were significantly higher in the third and fourth quartiles of daily salt intake than in the first quartile (odds ratios = 1.78 and 1.71, respectively). The present results suggest that the physical frailty status needs to be considered when providing salt reduction guidance to older adults for blood pressure control, in order to prevent progression of frailty and maintain quality of life.

**Figure:**
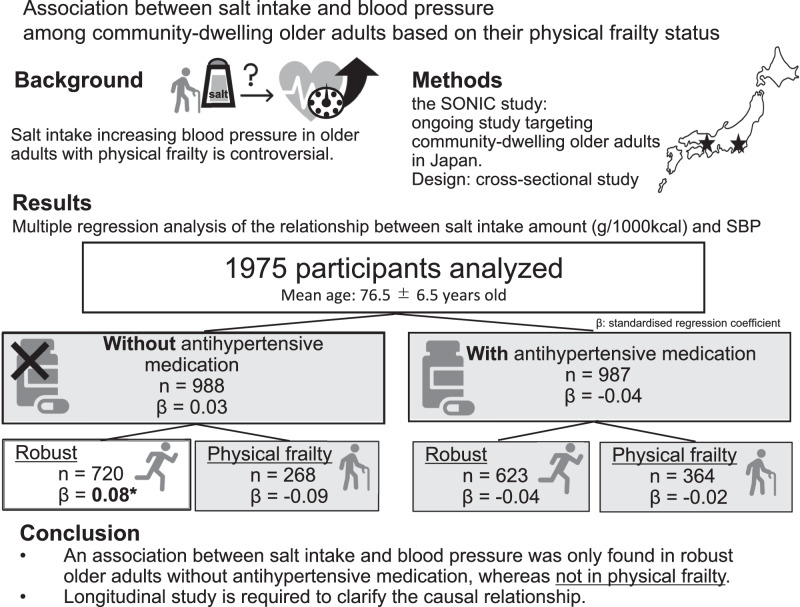
Cross-sectional analysis of the association between salt intake and blood pressure in community-dwelling older adults in Japan (SONIC study): the results suggest that salt intake may not be related to blood pressure in older adults with physical frailty.

Cross-sectional analysis of the association between salt intake and blood pressure in community-dwelling older adults in Japan (SONIC study): the results suggest that salt intake may not be related to blood pressure in older adults with physical frailty.

## Abstract (web summary)

Cross-sectional analysis of the association between salt intake and blood pressure in community-dwelling older adults in Japan (SONIC study): the results suggest that salt intake may not be related to blood pressure in older adults with physical frailty.

## Introduction

The percentage of the population aged 65 and older in Japan was 29.1% in 2023 [1], which is the highest worldwide [2]. The average life expectancy of people in Japan in 2022 was 81.0 years for males and 87.1 years for females [3]. Furthermore, the number of older adults requiring long-term care increased to 6.9 million in 2022 [4]. The main causes of long-term care in Japan are stroke due to hypertension and frailty due to old age [5]. Therefore, along with strategies to reduce the incidence of hypertension and its aggravation, improvements in physical function and the prevention of frailty are urgent issues.

The percentage of people with hypertension, the underlying cause of stroke [6, 7], is very high in Japan, and ~50% of people aged ≥70 years are currently taking antihypertensive medication [8]. Improvements in medication adherence and lifestyle modifications, such as salt reduction [9], are crucial for preventing the aggravation of hypertension. Frailty is a progressive age-related decline in physical systems that decreases the reserves of intrinsic capacity [10]. Among community-dwelling older adults aged ≥65 years in Japan, the prevalence of frailty was 8.7%, while that of prefrailty was 40.8% [11]. Since the percentage of older adults with frailty has markedly increased, effective measures based on evidence are needed. Adequate nutritional intake was previously shown to be critical for preventing frailty among older adults [12].

Therefore, salt reduction as blood pressure (BP) control to prevent stroke and adequate nutritional intake to prevent frailty need to be actively promoted in order to reduce the need for long-term care. However, with age-related changes in taste [13] and appetite decreases in older adults, extreme salt restriction may lead to nutritional deficiencies, including energy and protein, resulting in frailty [14]. In addition, meals are not only nutritional for older adults, the pleasure of eating appetizing food contributes to their quality of life [15]. Therefore, salt intake among older adults needs to be carefully managed to address both hypertension and the prevention of frailty.

Regarding the relationship between salt reduction and BP among older adults, a previous study showed that salt reduction effectively decreased BP [16]. However, another study demonstrated that a lower salt intake was associated with higher BP among community-dwelling older adults [17]. Therefore, the effects of salt intake on BP in older adults remain controversial. Accumulated evidence supporting salt reduction was primarily derived from study participants younger than 70 years [18], which is younger than the target age of people requiring protection against frailty. Moreover, the risk of mortality was higher among frail participants with a low salt intake than among robust participants [17]. Salt intake has been suggested to exert different effects on BP depending on the physical frailty status of older adults. To reduce the need for long-term care, further evidence is required to establish whether the effects of salt reduction on BP are dependent on the physical frailty status. Therefore, the present study investigated the association between daily salt intake and BP among community-dwelling older adults, including the very old people, based on their physical frailty status.

Point of view

- Clinical relevance
  In the context of the increasing hypertension patients with the onset of a super-aged society, managing blood pressure while considering diverse physical conditions such as frailty and comorbidities is becoming more important. Therefore, salt intake should be assessed individually bases, taking into account each person's nutritional status and comorbid conditions. Our findings demonstrating the association between salt intake and blood pressure based on the frailty status provide valuable insights for personalized salt-reduction guidance in the future.
- Future direction
  Further longitudinal research is required to examine the impact of salt intake on health outcomes in older adults to prevent the need for long-term care and maintain quality of life.
- Consideration for the Asian population
  Many Asian countries are experiencing rapid population aging and an expected increase in patients with hypertension and frailty status. For older adults, interventions must be tailored to individual physical conditions, with particular consideration given to physical frailty status and other health factors. This finding could be valuable in improving hypertension management in Asian countries with populations that have high salt intake.

## Methods

### Study design and setting

This was a cross-sectional analysis based on the Septuagenarians, Octogenarians, Nonagenarians Investigation with Centenarian’s (SONIC) study [19], an ongoing cohort study of community-dwelling older adults since 2010. Study participants were selected from Western Japan: Hyogo prefecture (rural: Asago city, urban: Itami city), and Eastern Japan: Tokyo prefecture (rural: Nishitama District, urban: Itabashi ward).

The SONIC study was approved by the Ethics Review Board of the Osaka University Graduate School of Medicine, Dentistry, and Human Sciences and the Tokyo Metropolitan Institute of Gerontology (approval numbers 266, H22-E9, 22 018, and 38, respectively). Written informed consent was obtained from all participants.

### Participants

Participants were randomly selected from the basic resident registration (*n* = 2245). Exclusion criteria for the present study were as follows: a lack of nutritional data (*n* = 10), the under- or over-reporting of energy (<600 kcal or >4000 kcal/day evaluated by a nutritional questionnaire) (*n* = 6), receiving dialysis (*n* = 1), and incomplete information on the variables examined (BP, blood test results, medications, smoking status, drinking status, physical measurements, and physical performance tests) (*n* = 253). In total, 1975 participants were included in the analysis.

### Daily salt and potassium intakes

Daily salt and potassium intakes were estimated by the brief-type self-administered diet history questionnaire (BDHQ). BDHQ is a dietary questionnaire that considers Japanese eating habits and obtains information on the consumption frequency of selected foods during the previous month to estimate the dietary intake of 58 food and beverage items [20]. BDHQ has a ranking ability for the intake of many nutrients [21] and its validity to ranking sodium and potassium has been shown, even in people >80 years [22]. Furthermore, the sodium intake estimated from BDHQ correlated with that measured by 24-h urine collection [23]. Participants completed BDHQ in advance and it was checked by trained survey staff at the venue. Daily salt and potassium intakes were adjusted for total energy intake by the density method [24] to obtain intake amounts per 1000 kcal.

### BP measurements and covariate data

We collected BP, medical, and physical data at the investigation venue.

BP was measured twice from both arms of participants in the sitting position after at least a few minutes of rest using a standard mercury sphygmomanometer by trained doctors or nurses. The mean value was adopted for BP [25]. Grip strength and gait speed were used to assess physical frailty. Grip strength was measured twice by the dominant hand while participants were seated with their arms held on their body using a Smedley hand dynamometer (Model YD-100; Yagami, Ltd., Nagoya, Japan). Gait speed was evaluated by the 8-feet gait speed test performed twice. Mean values were adopted for grip strength and gait speed. We defined physical frailty as grip strength <28 kg (male) or 18 kg (female) and gait speed <1.0 m/s [26], and higher values as robust.

Information on hypertensive medication was collected from medication notebooks and that on drinking and smoking statuses through an interview by trained medical doctors or nurses. We defined heavy drinkers as those who consumed 60 g or more of alcohol ≥3 times a week.

We measured height and weight, and calculated body mass index (BMI) as weight (kg)/height (m^2^). BMI ≥ 25 was defined as overweight [27]. Venous blood samples were collected to measure plasma glucose, creatinine, and other biochemical indicators. Diabetes mellitus (DM) was defined as casual plasma glucose ≥200 mg/dL, HbA1c ≥ 6.5%, or the current use of antidiabetic medication [28]. The estimated glomerular filtration rate (eGFR) was calculated by the equation of the Japanese Society of Nephrology using serum creatinine as follows: eGFR = 194 × Cr^−1.094^ × age^−0.287^ (×0.739 if female) [29]. Renal function decline was defined as eGFR < 60 mL/min/1.73 m^2^ [30].

Information was collected by medical professionals on the participant’s history of cerebrovascular disease and cardiovascular diseases. The BP measurement season (season of the survey date) was classified as winter (January and February), summer (July and August), and mid-term (March, September, October, and November).

### Statistical analysis

Descriptive data were presented as means and standard deviations or frequencies. The unpaired *t*-test and chi-square test were used to compare continuous and categorical variables, respectively. Robust and physical frailty groups were compared based on the use of antihypertensive medication. Participants were divided into quartiles according to their daily salt intake (g/1000 kcal), and the Jonckheere–Terpstra trend test and Cochran–Armitage test were then used to compare characteristics among the groups. A multiple regression analysis was performed to investigate the association between daily salt intake and BP. The dependent variables were systolic BP (SBP) or diastolic BP (DBP), while the independent variable was daily salt intake (g/1000 kcal). Since participants taking antihypertensive medication were considered to be treated with the goal of achieving BP < 140/90 mmHg, we conducted a logistic regression analysis. The dependent variables were SBP ≥ 140 mmHg or DBP ≥ 90 mmHg, while the independent variable was the daily salt intake quartile. The following covariates were examined: age group (70s, 80s, and 90s), sex, BMI, eGFR, DM, heavy drinking, smoking, daily potassium intake (mg/1000 kcal), physical frailty, and the use of antihypertensive medication, cerebrovascular disease and cardiovascular diseases, and measurement season. All participants were analyzed and were then divided into four groups based on the use of antihypertensive medication and their physical frailty status. The significance of differences was set at a *p* value < 0.05. Statistical analyses were performed using SPSS version 28 (IBM Japan, Tokyo, Japan) and JMP Pro ver.17 (SAS Institute, Cary, NC, USA).

## Results

The characteristics of participants are summarized in Table 1. Average age was 76.5 ± 6.5 years and 53.1% were female. The corresponding percentages of participants with physical frailty and taking antihypertensive medication were 32.0 and 50.0%, respectively. In a comparison based on the use of antihypertensive medication, the group taking antihypertensive medications had a higher mean age, mean SBP, percentage of participants with physical frailty, BMI ≥ 25, and eGFR < 60 mL/min/1.73 m^2^; mean daily salt intake (without antihypertensive medication: 6.4 g/1000 kcal, with antihypertensive medication: 6.4 g/1000 kcal) and potassium intake (without antihypertensive medication: 1605.2 mg/1000 kcal, with antihypertensive medication: 1590.2 mg/1000 kcal) did not significantly differ. Furthermore, no appreciable differences were observed in daily salt intake between the robust and physical frailty groups; however, daily potassium intake was consistently lower among the latter (without antihypertensive medication, robust: 1629.3 ± 408.1 mg, physical frailty: 1540.5 ± 388.6 mg, with antihypertensive medication, robust: 1614.4 ± 426.3, physical frailty: 1548.7 ± 414.3 mg). BP did not differ significantly between the two groups. BMI was lower in physical frailty participants regardless of the use of antihypertensive medication (without antihypertensive medication, robust: 22.2 ± 2.8 kg/m^2^, physical frailty: 21.4 ± 3.1 kg/m^2^, with antihypertensive medication, robust: 23.6 ± 2.9 kg/m^2^, physical frailty: 22.6 ± 3.3 kg/m^2^). The percentage of participants with eGFR < 60 mL/min/1.73 m^2^ was significantly higher among the physical frailty group than in the robust group (without antihypertensive medication, robust: 16.5%, physical frailty: 22.4%, with antihypertensive medication, robust: 30.0%, physical frailty: 41.8%).

**Table 1:** Characteristics of the study participants based on the use of antihypertensive medication and physical frailty status

	All	Without antihypertensive medication			With antihypertensive medication		
	*n* = 1975	Robust *n* = 720	Physical frailty *n* = 268	*p* value	Robust *n* = 623	Physical frailty *n* = 364	*p* value
Age, mean (SD)	76.5 (6.5)	73.7 (5.3)	79.2 (6.4)	<0.001	75.7 (5.7)	81.6 (6.5)	<0.001
Age group: 70s, *n* (%)	885 (44.8)	466 (64.7)	69 (25.7)	<0.001	296 (47.5)	54 (14.8)	<0.001
80s, *n* (%)	890 (45.1)	238 (33.1)	152 (56.7)		299 (48.0)	201 (55.2)	
90s, *n* (%)	200 (10.1)	16 (2.2)	47 (17.5)		28 (4.5)	109 (29.9)	
Female, *n* (%)	1049 (53.1)	384 (53.3)	159 (59.3)	0.092	301 (48.3)	205 (56.3)	0.015
Physical frailty, *n* (%)	632 (32.0)	–	–		–	–	
Grip strength (kg), mean (SD)	22.6 (8.3)	25.4 (7.9)	16.2 (5.6)	<0.001	25.6 (7.7)	16.7 (5.5)	<0.001
Gait time (s), mean (SD)	2.9 (1.3)	2.6 (0.6)	3.5 (2.1)	<0.001	2.7 (1.2)	3.6 (1.3)	<0.001
With antihypertensive medication, *n* (%)	987 (50.0)	–	–		–	–	
Daily salt intake (g/1000 kcal), mean (SD)	6.4 (1.3)	6.4 (1.3)	6.4 (1.4)	0.872	6.4 (1.3)	6.4 (1.4)	0.638
Daily potassium intake (mg/1000 kcal), mean (SD)	1598.1 (413.5)	1629.3 (408.1)	1540.5 (388.6)	0.002	1614.4 (426.3)	1548.7 (414.3)	0.018
Daily energy intake (kcal), mean (SD)	1941.4 (568.0)	1980.3 (565.7)	1896.8 (602.6)	0.043	1940.6 (563.8)	1898.5 (549.8)	0.253
Daily protein intake (% energy), mean (SD)	16.4 (3.3)	16.5 (3.2)	16.1 (3.2)	0.043	16.5 (3.2)	16.4 (3.6)	0.555
SBP (mmHg), mean (SD)	142.9 (18.7)	140.6 (19.6)	142.0 (21.1)	0.364	145.2 (16.3)	144.4 (18.3)	0.537
SBP ≥ 140 mmHg, *n* (%)	1084 (54.9)	348 (48.3)	137 (51.1)	0.436	383 (61.5)	216 (59.3)	0.507
DBP (mmHg), mean (SD)	77.8 (10.9)	78.7 (10.6)	76.1 (11.5)	0.001	79.0 (10.2)	75.2 (11.5)	0.537
DBP ≥ 90 mmHg, *n* (%)	258 (13.1)	104 (14.4)	25 (9.4)	0.035	91 (14.6)	38 (10.4)	0.061
BMI (kg/m^2^), mean (SD)	22.6 (3.0)	22.2 (2.8)	21.4 (3.1)	0.001	23.6 (2.9)	22.6 (3.3)	<0.001
BMI ≥ 25 kg/m^2^, *n* (%)	390 (19.7)	93 (12.9)	32 (11.9)	0.681	190 (30.5)	75 (20.6)	0.001
eGFR (mL/min/1.73 m^2^), mean (SD)	69.0 (16.2)	71.9 (13.3)	71.4 (16.8)	0.687	67.7 (16.4)	63.4 (19.0)	<0.001
eGFR < 60 mL/min/1.73 m^2^, *n* (%)	518 (26.2)	119 (16.5)	60 (22.4)	0.033	187 (30.0)	152 (41.8)	<0.001
DM, *n* (%)	344 (17.4)	98 (13.6)	51 (19.0)	0.034	113 (18.1)	82 (22.5)	0.095
Heavy drinking, *n* (%)	46 (2.3)	23 (3.2)	2 (0.7)	0.029	19 (3.0)	2 (0.5)	0.009
Smoking, *n* (%)	136 (6.9)	58 (8.1)	23 (8.6)	0.789	38 (6.1)	17 (4.7)	0.345
Cerebro and cardiovascular disease, *n* (%)	204 (10.3)	28 (3.9)	20 (7.5)	0.020	91 (14.6)	65 (17.9)	0.177
Season: Summer, *n* (%)	502 (25.4)	157 (21.8)	83 (31.0)	<0.001	150 (24.1)	112 (30.8)	0.003
Mid-term, *n* (%)	919 (46.5)	324 (45.0)	129 (48.1)		292 (46.9)	174 (47.8)	
Winter, *n* (%)	554 (28.1)	239 (33.2)	56 (20.9)		181 (29.1)	78 (21.4)	

Means (standard deviations) or frequencies are shown, as appropriate. The unpaired *t*-test and chi-square test and Cochran–Armitage test were used to compare continuous and categorical variables, respectively

*SBP* systolic blood pressure, *DBP* diastolic blood pressure, *BMI* body mass index, *eGFR* estimated glomerular filtration rate, *DM* diabetes mellitus

Table 2 shows the characteristics of the participants divided into quartiles based on daily salt intake (g/1000 kcal). A higher daily salt intake correlated with a higher daily potassium intake and a higher percentage of BMI ≥ 25. There was no clear association between the daily salt intake quartiles and physical frailty or antihypertensive medication use. When participants were divided by the use of antihypertensive medication and physical frailty (Supplementary Tables S2–S5), a higher daily salt intake was also associated with a higher daily potassium intake.

**Table 2:** Characteristics of the study participants by the daily salt intake (g/1000 kcal) quartile

	Daily salt intake (g/1000 kcal)				
	Q1 (*n* = 493)	Q2 (*n* = 495)	Q3 (*n* = 495)	Q4 (*n* = 494)	*p* value
	2.7–5.5 Mean: 4.8	5.4–6.3 Mean: 5.9	6.3–7.2 Mean: 6.7	7.2–15.6 Mean: 8.2	
Age, mean (SD)	76.7 (6.4)	76.3 (6.4)	76.8 (6.7)	76.5 (6.5)	0.925
Age group: 70s, *n* (%)	214 (43.4)	227 (45.9)	220 (44.6)	224 (45.3)	
80s, *n* (%)	229 (46.5)	224 (45.3)	214 (43.4)	223 (45.1)	
90s, *n* (%)	50 (10.1)	44 (8.9)	59 (12.0)	47 (9.5)	
Female, *n* (%)	225 (45.6)	260 (52.5)	285 (57.8)	279 (56.5)	<0.001
Physical frailty, *n* (%)	168 (34.1)	133 (26.9)	180 (36.5)	151 (30.6)	0.926
With antihypertensive medication, *n* (%)	249 (50.5)	233 (47.1)	250 (50.7)	255 (51.6)	0.487
Daily potassium intake (mg/1000 kcal), mean (SD)	1403.3 (400.8)	1550.6 (365.1)	1646.9 (371.0)	1789.9 (418.5)	<0.001
SBP ≥ 140 mmHg, *n* (%)	266 (54.0)	278 (56.2)	265 (53.8)	275 (55.7)	0.786
DBP ≥ 90 mmHg, *n* (%)	63 (12.8)	69 (13.9)	59 (12.0)	67 (13.6)	0.965
BMI ≥ 25 kg/m^2^, *n* (%)	81 (16.4)	89 (18.0)	105 (21.3)	115 (23.3)	0.003
eGFR < 60 mL/min/1.73 m^2^, *n* (%)	128 (26.0)	119 (24.0)	140 (28.4)	131 (26.5)	0.496
DM, *n* (%)	74 (15.0)	88 (17.8)	87 (17.6)	95 (19.2)	0.101
Heavy drinking, *n* (%)	23 (4.7)	10 (2.0)	7 (1.4)	6 (1.2)	<0.001
Smoking, *n* (%)	38 (7.7)	30 (6.1)	39 (7.9)	29 (5.9)	0.473
Cerebro and cardiovascular disease, *n* (%)	47 (9.5)	44 (8.9)	60 (12.2)	53 (10.7)	0.262
Season: Summer, *n* (%)	159 (32.3)	132 (26.7)	110 (22.3)	101 (20.4)	
Mid-term, *n* (%)	215 (43.6)	238 (48.1)	234 (47.5)	232 (47.0)	
Winter, *n* (%)	119 (24.1)	125 (25.3)	149 (30.2)	161 (32.6)	

Means (standard deviations) or frequencies are shown, as appropriate. The Jonckheere–Terpstra trend test and Cochran–Armitage test were used to compare continuous and categorical variables, respectively

*SBP* systolic blood pressure, *DBP* diastolic blood pressure, *BMI* body mass index, *eGFR* estimated glomerular filtration rate, *DM* diabetes mellitus

Table 3 shows the association between daily salt intake and BP. No correlations were observed among all participants or by the use of antihypertensive medication. However, in the analysis stratified by the use of antihypertensive medication and the physical frailty status, a correlation was noted between a higher daily salt intake (g/1000 kcal) and higher SBP (*β* = 0.08, *p* = 0.038) among the robust without antihypertensive medication group. A negative trend was also observed for potassium intake and SBP among this group. Similar results were obtained when we analyzed eGFR as a cut-off value of 60 mL/min/1.73 m^2^ rather than as a continuous variable (data were not shown). When we analyzed the subjects separately for the Western and Eastern regions, similar results were observed in the Western region (Supplementary Table S6). Moreover, among the robust without antihypertensive medication group, the odds ratio for SBP ≥ 140 mmHg was notably higher in the third and fourth quartiles of daily salt intake than in the first quartile (odds ratios = 1.78 and 1.71, respectively) (Table 4). No correlation was detected in participants with physical frailty or with the use of antihypertensive medication. Similarly, after adjusting for energy intake using the residual method for salt and potassium intake, the same trend was observed among robust without antihypertensive medication group (Supplementary Tables S7 and S8).

**Table 3:** Regression model for the association between daily salt or potassium intake (g/1000 kcal) and SBP or DBP

Study participants	Daily intake (/1000 kcal)			
	Salt (g)		Potassium (mg)	
	*β*	*p* value	*β*	*p* value
Dependent variable: SBP				
All^a^	0.00	0.972	−0.01	0.769
Without antihypertensive medication^b^	0.03	0.439	−0.05	0.192
Robust^c^	0.08	0.038	−0.07	0.077
Physical frailty^c^	−0.09	0.172	0.02	0.739
With antihypertensive medication^b^	−0.04	0.302	0.05	0.196
Robust^c^	−0.04	0.416	0.04	0.436
Physical frailty^c^	−0.02	0.761	0.06	0.346
Dependent variable: DBP				
All^a^	0.00	0.999	−0.04	0.106
Without antihypertensive medication^b^	0.02	0.630	−0.04	0.228
Robust^c^	0.05	0.253	−0.05	0.214
Physical frailty^c^	−0.04	0.527	−0.04	0.581
With antihypertensive medication^b^	−0.02	0.630	−0.03	0.421
Robust^c^	−0.03	0.445	−0.01	0.766
Physical frailty^c^	0.02	0.748	−0.05	0.353

*SBP* systolic blood pressure, *DBP* diastolic blood pressure, *β* standardized multi-regression coefficients

^a^Adjusted with age group, sex, BMI, eGFR, DM, heavy drinking, smoking, physical frailty, antihypertensive medication, cardiovascular disease and cerebrovascular disease, season

^b^Adjusted with age group, sex, BMI, eGFR, DM, heavy drinking, smoking, physical frailty, cardiovascular disease and cerebrovascular disease, season

^c^Adjusted with age group, sex, BMI, eGFR, DM, heavy drinking, smoking, cardiovascular disease and cerebrovascular disease, season

**Table 4:** Odds ratio and 95% confidence intervals for the daily salt intake (g/1000 kcal) quartiles and SBP ≥ 140 mmHg or DBP ≥ 90 mmHg calculated by a logistic regression model

Study participants	Daily salt intake amount (g/1000 kcal)						
	Q1	Q2		Q3		Q4	
	OR (95%CI)	OR (95%CI)	*p* value	OR (95%CI)	*p* value	OR (95%CI)	*p* value
Dependent variable: SBP ≥ 140 mmHg							
All participants^a^	ref.	1.14 (0.88–1.48)	0.326	1.05 (0.81–1.38)	0.702	1.11 (0.84–1.46)	0.451
Without antihypertensive medication^b^	ref.	1.03 (0.71–1.49)	0.874	1.24 (0.85–1.82)	0.262	1.28 (0.86–1.89)	0.222
Robust^c^	ref.	1.50 (0.97–2.33)	0.071	1.78 (1.13–2.81)	0.013	1.71 (1.07–2.74)	0.024
Physical frailty^c^	ref.	0.53 (0.25–1.13)	0.099	0.61 (0.29–1.31)	0.206	0.81 (0.37–1.74)	0.585
With antihypertensive medication^b^	ref.	1.34 (0.92–1.96)	0.133	0.94 (0.64–1.38)	0.763	0.94 (0.63–1.39)	0.753
Robust^c^	ref.	1.38 (0.84–2.25)	0.199	0.83 (0.50–1.35)	0.448	0.85 (0.51–1.41)	0.536
Physical frailty^c^	ref.	0.97 (0.51–1.82)	0.915	1.36 (0.72–2.57)	0.341	1.15 (0.59–2.22)	0.686
Dependent variable: DBP ≥ 90 mmHg							
All participants^a^	ref.	1.14 (0.78–1.66)	0.491	1.05 (0.70–1.56)	0.817	1.20 (0.80–1.79)	0.375
Without antihypertensive medication^b^	ref.	1.14 (0.66–1.99)	0.637	1.01 (0.57–1.82)	0.965	1.52 (0.86–2.7)	0.147
Robust^c^	ref.	1.66 (0.88–3.12)	0.115	1.26 (0.64–2.50)	0.506	1.84 (0.93–3.65)	0.080
Physical frailty^c^	ref.	0.34 (0.08–1.40)	0.137	0.28 (0.07–1.14)	0.076	1.04 (0.36–2.98)	0.945
With antihypertensive medication^b^	ref.	1.21 (0.72–2.02)	0.478	0.91 (0.52–1.61)	0.754	0.90 (0.50–1.61)	0.729
Robust^c^	ref.	0.89 (0.48–1.65)	0.716	0.57 (0.28–1.16)	0.123	0.73 (0.37–1.44)	0.361
Physical frailty^c^	ref.	2.03 (0.71–5.84)	0.189	1.90 (0.67–5.39)	0.227	1.09 (0.33–3.63)	0.887

*OR* odds ratio, *CI* confidence interval, *BP* blood pressure

^a^Adjusted with age group, sex, BMI ≥ 25, eGFR < 60, DM, heavy drinking, smoking, the daily potassium intake (mg/1000 kcal) quartile, physical frailty, antihypertensive medication, cardiovascular disease and cerebrovascular disease, season

^b^Adjusted with age group, sex, BMI ≥ 25, eGFR < 60, DM, heavy drinking, smoking, the daily potassium intake (mg/1000 kcal) quartile, physical frailty, cardiovascular disease and cerebrovascular disease, season

^c^Adjusted with age group, sex, BMI ≥ 25, eGFR < 60, DM, heavy drinking, smoking, the daily potassium intake (mg/1000 kcal) quartile, cardiovascular disease and cerebrovascular disease, season

## Discussion

We investigated the association between salt intake and BP among community-dwelling older adults based on their physical frailty status. The results obtained confirmed the association between high salt intake and high SBP among the robust without antihypertensive medication group only. It is important to note this association was not observed among all participants, or the physical frailty group, or the use of antihypertensive medication group. The association between salt intake and BP among the older adults including very old people, with a focus on the physical frailty status is highly novel.

Previous studies have reported that BP increases with age [31, 32]. Despite the older age of participants in the present study and their presumed salt sensitivity, a clear association between salt intake and BP was not found among all participants nor in the physical frailty group. This may be attributed to the target age being older and the associated characteristics of physical frailty. The mechanisms by which salt intake did not notably affect BP in older adults with physical frailty may be due to the impaired regulation of the autonomic nervous system [33] and advanced arterial stiffness [34, 35]. In other words, the response to salt intake appeared to vary among older adults with physical frailty because of the large individual variability in their physical condition. Therefore, no consistent trend was observed in the analysis at the group level. These results suggest that the effects of salt intake on BP were not substantial among older adults with physical frailty. In addition, the daily potassium intake and BMI were lower among the physical frailty group than among the robust group. Older adults in this group may have difficulty maintaining an adequate quantity and balanced diet. Therefore, adequate nutritional intake may need to be prioritized over salt reduction in the management of hypertension for older adults with physical frailty.

Among the robust without antihypertensive medication group, associations were observed between salt intake and SBP, and there was a tendency for a negative correlation with potassium. This result was consistent with previous findings showing higher BP with a higher salt intake or sodium-to-potassium ratio [36, 37]. A high salt intake and elevated BP are due to a number of factors, including water retention and an increase in systemic peripheral resistance [38]. The present study suggests that this mechanism may have worked in healthy older adults. When examined by region, this trend was particularly pronounced in the Western area, suggesting that there may be unaccounted factors, such as differing dietary habits, that could contribute to these regional variations.

On the other hand, no clear association was noted between salt intake and BP among the robust with antihypertensive medication group. In the present study, the effect of antihypertensive medication might have been greater than that of daily salt intake for BP. In patients taking antihypertensive medication, it is plausible that the type and dosage of the medication may vary based on their physical frailty status. Consequently, it is suggested that no association was observed between salt intake and BP, regardless of frailty status, in participants using antihypertensive medications. In addition, since the Japanese Guidelines for the Treatment of Hypertension stipulate salt reduction in hypertensive subjects receiving antihypertensive treatment [39], we cannot rule out the possibility that the participants had already decreased their salt intake while receiving treatment.

There are several limitations that need to be addressed. First, daily salt intake was not measured by 24-h urine collection, which is the gold standard for estimations of salt intake. Although the estimated salt intake from the BDHQ correlated with the values calculated using the dietary record method [22], accurately estimating the actual intake remains difficult, and the results did not indicate a specific salt intake.　However, BDHQ, which was used in the present study, asks about dietary intakes during the preceding month, allowing us to take into account diurnal variations in daily salt intake. Second, we measured BP at the venue, not home BP, which is considered to be more stable for an accurate evaluation of BP. Nevertheless, measuring BP at the venue allowed the collection of BP data from a large number of community-dwelling older adults, including those aged 90 years. Third, the type and amount of antihypertensive medication was not considered in the present study. Fourth, although Fried’s frailty phenotype criteria [40] are commonly employed to assess frailty, this study utilized walking speed and grip strength as the indicators. However, the combination of walking speed and grip strength demonstrated greater accuracy, precision, specificity, and sensitivity compared to other potential combinations within Fried’s frailty phenotype [41]. Therefore, it is regarded as a valuable tool for conveniently predicting physical frailty. Future research should consider incorporating additional components to enable a more comprehensive assessment. Finally, since this study had a cross-sectional design, it was not possible to prove a causal relationship. Therefore, longitudinal research is required.

### Perspective of Asia

Many Asian countries are experiencing rapid population aging [2] and an expected increase in patients with hypertension and frailty status. Therefore, the findings of this study may contribute to maintain and improve QOL of older adults in Asian countries.

## Conclusion

The present results showed that among community-dwelling older adults, an association was observed between salt intake and SBP in those who were robust and not taking antihypertensive medication, but not in those with physical frailty. Therefore, consideration of the physical frailty status is required when providing salt restriction guidance to older adults for the prevention of hypertension and its aggravation, and, if necessary, adequate nutritional intake needs to be prioritized in order to prevent the progression of frailty and maintain quality of life. However, longitudinal research is warranted to clarify the causal relationship between daily salt intake and BP among community-dwelling older adults.

## Supplementary information

Supplementary information
