# Supplementary material for: Association between salt intake and blood pressure among community-dwelling older adults based on their physical frailty status
Source: Hypertens Res. 2025 Jan 17;48(4):1399–408. doi: 10.1038/s41440-024-02066-y (PMC11972958; doi:10.1038/s41440-024-02066-y)
Supplement: Supplementary file 1 — Supplementary information [file 41440_2024_2066_MOESM1_ESM.docx]

Table S1 Characteristics of the study participants based on the use of antihypertensive medication and physical frailty status

|  | antihypertensive medication | |  | Physical frailty status | | |
| --- | --- | --- | --- | --- | --- | --- |
|  | Without | With |  | Robust | Physical frailty |  |
|  | n=988 | n=987 | p-value | n=1343 | n=632 | p-value |
| Age, mean (SD) | 75.2(6.1) | 77.9(6.7) | <0.001 | 74.6(5.6) | 80.6(6.6) | <0.001 |
| Age group: 70s, n (%) | 535(54.1) | 350(35.5) | <0.001 | 762(56.7) | 123(19.5) |  |
| 80s, n (%) | 390(39.5) | 500(50.7) |  | 537(40) | 353(55.9) |  |
| 90s, n (%) | 63(6.4) | 137(13.9) |  | 44(3.3) | 156(24.7) |  |
| Female, n (%) | 543(55.0) | 506(51.3) | 0.100 | 685(51.0) | 364(57.6) | 0.006 |
| Physical frailty, n (%) | 268(27.1) | 364(36.9) | <0.001 | - | - |  |
| Grip strength (kg), mean (SD) | 22.9(8.4) | 22.4(8.2) | 0.165 | 25.5(7.8) | 16.5(5.5) | <0.001 |
| Gait time (s), mean (SD) | 2.8(1.3) | 3.0(1.3) | 0.002 | 2.6(1.0) | 3.5(1.7) | <0.001 |
| Daily salt intake  (g/1000 kcal), mean (SD) | 6.4(1.3) | 6.4(1.4) | 0.507 | 6.4(1.3) | 6.4(1.4) | 0.699 |
| Daily potassium intake  (mg/1000 kcal), mean (SD) | 1605.2(404.6) | 1590.2(422.9) | 0.421 | 1622.4(416.6) | 1545.2(403.3) | <0.001 |
| Daily energy intake (kcal), mean (SD) | 1957.6(576.8) | 1925.1(558.8) | 0.203 | 1961.9(564.9) | 1897.8(572.3) | 0.019 |
| Daily protein intake (% energy), mean (SD) | 16.4(3.2) | 16.4(3.4) | 0.732 | 16.5(3.2) | 16.2(3.5) | 0.091 |
| SBP (mmHg), mean (SD) | 141.0(20.0) | 144.9(17.0) | <0.001 | 142.7(18.3) | 143.4(19.6) | 0.470 |
| SBP ≥ 140 mmHg, n (%) | 485(49.1) | 599(60.7) | <0.001 | 731(54.4) | 353(55.9) | 0.553 |
| DBP (mmHg), mean (SD) | 78.0(10.9) | 77.6(10.8) | 0.420 | 78.8(10.4) | 75.6(11.5) | <0.001 |
| DBP ≥ 90 mmHg, n (%) | 129(13.1) | 129(13.1) | 1.000 | 195(14.5) | 63(10.0) | 0.005 |
| BMI (kg/m^2^), mean (SD) | 22.0(2.9) | 23.2(3.1) | <0.001 | 22.8(2.9) | 22.1(3.2) | <0.001 |
| BMI ≥ 25 kg/m^2^, n (%) | 125(12.7) | 265(26.8) | <0.001 | 283(21.1) | 107(16.9) | 0.031 |
| eGFR (mL/min/1.73 m^2^), mean (SD) | 71.8(14.3) | 66.1(17.5) | <0.001 | 70(14.9) | 66.8(18.5) | <0.001 |
| eGFR < 60 mL/min/1.73 m^2^, n (%) | 179(18.1) | 339(34.3) | <0.001 | 306(22.8) | 212(33.5) | 0.000 |
| DM, n (%) | 149(15.1) | 195(19.8) | 0.006 | 211(15.7) | 133(21.0) | 0.004 |
| Heavy drinking, n (%) | 25(2.5) | 21(2.1) | 0.553 | 42(3.1) | 4(0.6) | 0.001 |
| Smoking, n (%) | 81(8.2) | 55(5.6) | 0.021 | 96(7.1) | 40(6.3) | 0.503 |
| Cerebro & Cardio vascular disease, n (%) | 48(4.9) | 156(15.8) | <0.01 | 119(8.9) | 85(13.4) | 0.002 |
| Season: Summer, n (%) | 240(24.3) | 262(26.5) | 0.074 | 307(22.9) | 195(30.9) | <0.001 |
| Mid-term, n (%) | 453(45.9) | 466(47.2) |  | 616(45.9) | 303(47.9) |  |
| Winter, n (%) | 295(29.9) | 259(26.2) |  | 420(31.3) | 134(21.2) |  |

Means (standard deviations) or frequencies are shown, as appropriate. The unpaired t-test and chi-square test and Cochran-Armitage test were used to compare continuous and categorical variables, respectively.

*SBP* systolic blood pressure, *DBP* diastolic blood pressure, *BMI* body mass index, *eGFR* estimated glomerular filtration rate, *DM* diabetes mellitus

Table S2 Characteristics of the study participants (robust without antihypertensive medication) by the daily salt intake (g/1000 kcal) quartile

|  | Daily salt intake amount (g/1,000 kcal) | | | | |
| --- | --- | --- | --- | --- | --- |
|  | Q1 (n = 180) | Q2 (n = 180) | Q3 (n = 180) | Q4 (n = 180) |  |
|  | 2.8-5.5  mean: 4.9* | 5.5-6.2  mean: 5.9 | 6.2-7.2  mean:6.7 | 7.2-12.2  mean:8.1 | p-value |
| Age, mean (SD) | 73.9(5.4) | 74.0(5.5) | 73.4(4.7) | 73.7(5.5) | 0.791 |
| Age group: 70s, n (%) | 114(63.3) | 113(62.8) | 120(66.7) | 119(66.1) |  |
| 80s, n (%) | 61(33.9) | 61(33.9) | 60(33.3) | 56(31.1) |  |
| 90s, n (%) | 5(2.8) | 6(3.3) | 0(0.0) | 5(2.8) |  |
| Female, n (%) | 78(43.3) | 93(51.7) | 107(59.4) | 106(58.9) | 0.001 |
| Daily potassium intake  (mg/1000 kcal), mean (SD) | 1434.4(393.6) | 1549.6(367.3) | 1677.1(358.2) | 1856.1(390.2) | <0.001 |
| SBP ≥ 140 mmHg, n (%) | 76(42.2) | 89(49.4) | 92(51.1) | 91(50.6) | 0.110 |
| DBP ≥ 90 mmHg, n (%) | 20(11.1) | 30(16.7) | 24(13.3) | 30(16.7) | 0.255 |
| BMI ≥ 25 kg/m^2^, n (%) | 22(12.2) | 24(13.3) | 21(11.7) | 26(14.4) | 0.655 |
| eGFR < 60 mL/min/1.73 m^2^, n (%) | 29(16.1) | 32(17.8) | 32(17.8) | 26(14.4) | 0.686 |
| DM, n (%) | 22(12.2) | 27(15.0) | 28(15.6) | 21(11.7) | 0.923 |
| Heavy drinking, n (%) | 12(6.7) | 5(2.8) | 2(1.1) | 4(2.2) | 0.011 |
| Smoking, n (%) | 16(8.9) | 13(7.2) | 19(10.6) | 10(5.6) | 0.462 |
| Cerebro & Cardio vascular disease, n (%) | 6(3.3) | 5(2.8) | 9(5.0) | 8(4.4) | 0.389 |
| Season: Summer, n (%) | 60(33.3) | 38(21.1) | 33(18.3) | 26(14.4) |  |
| Mid-term, n (%) | 70(38.9) | 93(51.7) | 82(45.6) | 79(43.9) |  |
| Winter, n (%) | 50(27.8) | 49(27.2) | 65(36.1) | 75(41.7) |  |

Means (standard deviations) or frequencies are shown, as appropriate. The Jonckheere-Terpstra trend test and Cochran-Armitage test were used to compare continuous and categorical variables, respectively.

*SBP* systolic blood pressure, *DBP* diastolic blood pressure, *BMI* body mass index, *eGFR* estimated glomerular filtration rate, *DM*: diabetes mellitus.

Table S3 Characteristics of the study participants (physical frailty without antihypertensive medication) by the daily salt intake (g/1000 kcal) quartile

|  | Daily salt intake amount (g/1000 kcal) | | | | |
| --- | --- | --- | --- | --- | --- |
|  | Q1 (n = 67) | Q2 (n = 67) | Q3 (n = 67) | Q4 (n = 67) |  |
|  | 2.7-5.4  mean: 4.7 | 5.4-6.3  mean:6.0 | 6.3-7.1  mean:6.7 | 7.1-12.4  mean:8.1 | p-value |
| Age, mean (SD) | 78.4(6.3) | 79.4(6.4) | 79.4(7.1) | 79.6(5.9) | 0.288 |
| Age group: 70s, n (%) | 20(29.9) | 16(23.9) | 19(28.4) | 14(20.9) |  |
| 80s, n (%) | 38(56.7) | 39(58.2) | 33(49.3) | 42(62.7) |  |
| 90s, n (%) | 9(13.4) | 12(17.9) | 15(22.4) | 11(16.4) |  |
| Female, n (%) | 37(55.2) | 39(58.2) | 45(67.2) | 38(56.7) | 0.617 |
| Daily potassium intake  (mg/1000 kcal), mean (SD) | 1400.4(411) | 1562.6(348.4) | 1587.0(334.8) | 1611.9(425) | <0.001 |
| SBP ≥ 140 mmHg, n (%) | 39(58.2) | 28(41.8) | 32(47.8) | 38(56.7) | 0.956 |
| DBP ≥ 90 mmHg, n (%) | 9(13.6) | 3(4.5) | 3(4.5) | 10(14.9) | 0.798 |
| BMI ≥ 25 kg/m^2^, n (%) | 8(11.9) | 1(1.5) | 12(17.9) | 11(16.4) | 0.092 |
| eGFR < 60 mL/min/1.73 m^2^, n (%) | 12(17.9) | 17(25.4) | 15(22.4) | 16(23.9) | 0.512 |
| DM, n (%) | 13(19.4) | 12(17.9) | 14(20.9) | 12(17.9) | 0.945 |
| Heavy drinking, n (%) | 2(3.0) | 0(0.0) | 0(0.0) | 0(0.0) | 0.057 |
| Smoking, n (%) | 6(9.0) | 3(4.5) | 6(9.0) | 8(11.9) | 0.380 |
| Cerebro & Cardio vascular disease, n (%) | 3(4.5) | 6(9.0) | 7(10.4) | 4(6.0) | 0.678 |
| Season: Summer, n (%) | 24(35.8) | 22(32.8) | 17(25.4) | 20(29.9) |  |
| Mid-term, n (%) | 30(44.8) | 26(38.8) | 38(56.7) | 35(52.2) |  |
| Winter, n (%) | 13(19.4) | 19(28.4) | 12(17.9) | 12(17.9) |  |

Means (standard deviations) or frequencies are shown, as appropriate. The Jonckheere-Terpstra trend test and Cochran-Armitage test were used to compare continuous and categorical variables, respectively.

*SBP* systolic blood pressure, *DBP* diastolic blood pressure, *BMI* body mass index, *eGFR* estimated glomerular filtration rate, *DM*: diabetes mellitus.

Table S4 Characteristics of the study participants (robust with antihypertensive medication) by the daily salt intake (g/1000 kcal) quartile

|  | Daily salt intake amount (g/1000 kcal) | | | | |
| --- | --- | --- | --- | --- | --- |
|  | Q1 (n=155) | Q2 (n=156) | Q3 (n=156) | Q4 (n=156) |  |
|  | 3.3-5.5  mean: 4.9 | 5.5-6.3  mean: 5.9 | 6.3-7.2  mean: 6.7 | 7.2-15.6  mean:8.2 | p-value |
| Age, mean (SD) | 75.7(5.8) | 75.7(5.9) | 76.0(5.9) | 75.4(5.5) | 0.527 |
| Age group: 70s, n (%) | 74(47.7) | 77(49.4) | 71(45.5) | 74(47.4) |  |
| 80s, n (%) | 73(47.1) | 71(45.5) | 77(49.4) | 78(50) |  |
| 90s, n (%) | 8(5.2) | 8(5.1) | 8(5.1) | 4(2.6) |  |
| Female, n (%) | 63(40.6) | 74(47.4) | 84(53.8) | 80(51.3) | 0.033 |
| Daily potassium intake  (mg/1000 kcal), mean (SD) | 1407.9(429) | 1555.2(372.9) | 1690.6(349.3) | 1802.7(444.7) | <0.001 |
| SBP ≥ 140 mmHg, n (%) | 96(61.9) | 108(69.2) | 88(56.4) | 91(58.3) | 0.175 |
| DBP ≥ 90 mmHg, n (%) | 28(18.1) | 26(16.7) | 16(10.3) | 21(13.5) | 0.111 |
| BMI ≥ 25 kg/m^2^, n (%) | 36(23.2) | 53(34.0) | 48(30.8) | 53(34.0) | 0.079 |
| eGFR < 60 mL/min/1.73 m^2^, n (%) | 49(31.6) | 48(30.8) | 46(29.5) | 44(28.2) | 0.484 |
| DM, n (%) | 18(11.6) | 29(18.6) | 28(17.9) | 38(24.4) | 0.007 |
| Heavy drinking, n (%) | 9(5.8) | 5(3.2) | 4(2.6) | 1(0.6) | 0.009 |
| Smoking, n (%) | 11(7.1) | 8(5.1) | 10(6.4) | 9(5.8) | 0.754 |
| Cerebro & Cardio vascular disease, n (%) | 19(12.3) | 22(14.1) | 23(14.7) | 27(17.3) | 0.212 |
| Season: Summer, n (%) | 44(28.4) | 39(25) | 35(22.4) | 32(20.5) |  |
| Mid-term, n (%) | 69(44.5) | 78(50) | 72(46.2) | 73(46.8) |  |
| Winter, n (%) | 42(27.1) | 39(25) | 49(31.4) | 51(32.7) |  |

Means (standard deviations) or frequencies are shown, as appropriate. The Jonckheere-Terpstra trend test and Cochran-Armitage test were used to compare continuous and categorical variables, respectively.

*SBP* systolic blood pressure, *DBP* diastolic blood pressure, *BMI* body mass index, *eGFR* estimated glomerular filtration rate, *DM*: diabetes mellitus.

Table S5 Characteristics of study participants (physical frailty with antihypertensive medication) by the daily salt intake (g/1000kcal) quartile

|  | Daily salt intake amount (g/1000kcal) | | | | |
| --- | --- | --- | --- | --- | --- |
|  | Q1 (n = 91) | Q2 (n = 91) | Q3 (n = 91) | Q4 (n = 91) |  |
|  | 3.6-5.3  mean: 4.7 | 5.4-6.3  mean: 5.9 | 6.3-7.2  mean: 6.7 | 7.2-12.3  mean: 8.2 | p-value |
| Age, mean (SD) | 81.6(6.1) | 81.9(6.0) | 81.3(7.1) | 81.4(6.8) | 0.759 |
| Age group: 70s, n (%) | 11(12.1) | 9(9.9) | 18(19.8) | 16(17.6) |  |
| 80s, n (%) | 54(59.3) | 55(60.4) | 44(48.4) | 48(52.7) |  |
| 90s, n (%) | 26(28.6) | 27(29.7) | 29(31.9) | 27(29.7) |  |
| Female, n (%) | 43(47.3) | 52(57.1) | 54(59.3) | 56(61.5) | 0.053 |
| Daily potassium intake  (mg/1000 kcal), mean (SD) | 1321.1(336.4) | 1541.2(367.2) | 1582(434.7) | 1750.3(401.0) | <0.001 |
| SBP ≥ 140 mmHg, n (%) | 51(56.0) | 54(59.3) | 56(61.5) | 55(60.4) | 0.504 |
| DBP ≥ 90 mmHg, n (%) | 7(7.7) | 12(13.2) | 12(13.2) | 7(7.7) | 1.000 |
| BMI ≥ 25 kg/m^2^, n (%) | 16(17.6) | 12(13.2) | 21(23.1) | 26(28.6) | 0.024 |
| eGFR < 60 mL/min/1.73 m^2^, n (%) | 34(37.4) | 32(35.2) | 42(46.2) | 44(48.4) | 0.057 |
| DM, n (%) | 18(19.8) | 22(24.2) | 19(20.9) | 23(25.3) | 0.501 |
| Heavy drinking, n (%) | 0(0.0) | 0(0.0) | 1(1.1) | 1(1.1) | 0.205 |
| Smoking, n (%) | 6(6.6) | 4(4.4) | 5(5.5) | 2(2.2) | 0.222 |
| Cerebro & Cardio vascular disease, n (%) | 17(18.7) | 16(17.6) | 18(19.8) | 14(15.4) | 0.668 |
| Season: Summer, n (%) | 31(34.1) | 36(39.6) | 21(23.1) | 24(26.4) |  |
| Mid-term, n (%) | 45(49.5) | 39(42.9) | 46(50.5) | 44(48.4) |  |
| Winter, n (%) | 15(16.5) | 16(17.6) | 24(26.4) | 23(25.3) |  |

Means (standard deviations) or frequencies are shown, as appropriate. The Jonckheere-Terpstra trend test and Cochran-Armitage test were used to compare continuous and categorical variables, respectively.

*SBP* systolic blood pressure, *DBP* diastolic blood pressure, *BMI* body mass index, *eGFR* estimated glomerular filtration rate, *DM*: diabetes mellitus.

|  | Western  n=935 | | Eastern  n=1040 | |
| --- | --- | --- | --- | --- |
| Study participants | β | p-value | β | p-value |
| Dependent variable: SBP |  |  |  |  |
| All _a_ | -0.02 | 0.515 | 0.01 | 0.717 |
| Without antihypertensive medication _b_ | 0.027 | 0.583 | 0.03 | 0.454 |
| Robust _c_ | 0.13 | 0.031 | 0.06 | 0.297 |
| Physical frailty _c_ | -0.13 | 0.141 | 0.01 | 0.883 |
| With antihypertensive medication _b_ | -0.10 | 0.067 | -0.01 | 0.885 |
| Robust _c_ | -0.17 | 0.010 | 0.04 | 0.463 |
| Physical frailty _c_ | 0.01 | 0.943 | -0.06 | 0.429 |
| Dependent variable: DBP |  |  |  |  |
| All _a_ | 0.01 | 0.872 | 0.00 | 0.999 |
| Without antihypertensive medication _b_ | 0.07 | 0.168 | 0.00 | 0.997 |
| Robust _c_ | 0.12 | 0.040 | 0.01 | 0.859 |
| Physical frailty _c_ | -0.02 | 0.793 | -0.06 | 0.526 |
| With antihypertensive medication _b_ | -0.07 | 0.150 | 0.01 | 0.760 |
| Robust _c_ | -0.10 | 0.162 | 0.00 | 0.947 |
| Physical frailty _c_ | -0.05 | 0.562 | 0.04 | 0.588 |

Table S6 Regression model for the association between the daily salt intake (g/1000 kcal) and SBP or DBP by area

a: Adjusted with age group, sex, BMI, eGFR, DM, heavy drinking, smoking, daily potassium intake (mg/1000 kcal), physical frailty, antihypertensive medication, cardiovascular disease and cerebrovascular disease, season

b: Adjusted with, age group, sex, BMI, eGFR, DM, heavy drinking, smoking, daily potassium intake (mg/1000 kcal), physical frailty, cardiovascular disease and cerebrovascular disease, season

c: Adjusted with age group, sex, BMI, eGFR, DM, heavy drinking, smoking, daily potassium intake (mg/1000 kcal), cardiovascular disease and cerebrovascular disease, season

*SBP* systolic blood pressure, *DBP* diastolic blood pressure, *β* standardized multi-regression coefficients

|  | Daily intake | | | | |
| --- | --- | --- | --- | --- | --- |
|  | Salt (g) | |  | Potassium (mg) | |
| Study participants | β | p-value |  | β | p-value |
| Dependent variable: SBP |  |  |  |  |  |
| Without antihypertensive medication |  |  |  |  |  |
| Robust | 0.07 | 0.069 |  | -0.07 | 0.095 |
| Physical frailty | 0.08 | 0.247 |  | 0.02 | 0.736 |
| With antihypertensive medication |  |  |  |  |  |
| Robust _c_ | -0.03 | 0.458 |  | 0.03 | 0.526 |
| Physical frailty _c_ | -0.03 | 0.542 |  | 0.06 | 0.296 |
| Dependent variable: DBP |  |  |  |  |  |
| Without antihypertensive medication |  |  |  |  |  |
| Robust | 0.03 | 0.398 |  | -0.04 | 0.371 |
| Physical frailty | -0.03 | 0.683 |  | -0.01 | 0.822 |
| With antihypertensive medication |  |  |  |  |  |
| Robust | -0.04 | 0.332 |  | 0.01 | 0.813 |
| Physical frailty | 0.02 | 0.698 |  | -0.05 | 0.422 |

Table S7 Regression model for the association between daily salt or potassium intake (adjusted by residual method) and SBP or DBP

Adjusted with age group, sex, BMI, eGFR, DM, heavy drinking, smoking, cardiovascular disease and cerebrovascular disease, season

*SBP* systolic blood pressure, *DBP* diastolic blood pressure, *β* standardized multi-regression coefficients

Table S8 Odds ratio and 95% confidence intervals for the daily salt intake (adjusted by residual method) quartiles and BP ≥ 140/ 90 mmHg calculated by logistic regression model

| Study participants | Daily salt intake amount (g) | | | | | | |
| --- | --- | --- | --- | --- | --- | --- | --- |
|  | Q1 | Q2 | | Q3 | | Q4 | |
|  | OR (95%CI) | OR (95%CI) | p-value | OR (95%CI) | p-value | OR (95%CI) | p-value |
| Without antihypertensive medication |  |  |  |  |  |  |  |
| Robust | ref. | 1.32(0.85-2.05) | 0.215 | 1.63(1.03-2.57) | 0.035 | 1.79(1.12-2.85) | 0.015 |
| Physical frailty | ref. | 0.33(0.15-0.72) | 0.005 | 0.48(0.22-1.05) | 0.067 | 0.59(0.27-1.29) | 0.185 |
| With antihypertensive medication |  |  |  |  |  |  |  |
| Robust | ref. | 1.27(0.78-2.09) | 0.337 | 0.76(0.47-1.24) | 0.271 | 0.99(0.59-1.66) | 0.965 |
| Physical frailty | ref. | 0.78(0.41-1.49) | 0.456 | 1.78(0.92-3.44) | 0.086 | 1.14(0.58-2.23) | 0.709 |

Adjusted with age group, sex, BMI ≥ 25, eGFR < 60, DM, heavy drinking, smoking, the daily potassium intake (mg) quartile, cardiovascular disease and cerebrovascular disease, season

*OR* odds ratio, *CI* confidence interval, *BP* blood pressure
